# Medical Residency Admissions in India and the United States: A Comparative Analysis of Challenges and Reforms

**DOI:** 10.7759/cureus.81553

**Published:** 2025-04-01

**Authors:** Nirupam Nadella, Gayatri R Chagamreddy, Lokesh Edara, Korvi N Kumar, Vaishnavi Yadla

**Affiliations:** 1 Department of General Medicine, Dr. D.Y. Patil Medical College Hospital and Research Centre, Dr. D.Y. Patil Vidyapeeth (Deemed to Be University), Pune, IND; 2 Department of Internal Medicine, Western Michigan University, Kalamazoo, USA; 3 Department of General Medicine, Apollo Institute of Medical Sciences and Research, Chittoor, IND

**Keywords:** admission challenges and reforms, healthcare workforce training, medical residency admissions, medical school to residency transition, nrmp match system, postgraduate medical counseling, seat allocation in medical residency

## Abstract

The process of medical residency admissions varies significantly between India and the United States, presenting distinct challenges and reforms in each system. India's postgraduate medical entrance has undergone major transformations, consolidating multiple exams into the National Eligibility Cum Entrance Test Post Graduate (NEET PG) to create a unified admission process. However, asynchronous counseling rounds, seat blocking, and judicial interventions continue to plague the system, resulting in inefficiencies and inequities. In contrast, the U.S. employs a structured and algorithm-driven residency matching system, primarily managed by the National Resident Matching Program (NRMP) and specialized match programs like the San Francisco Match and the Urology Match. The NRMP's "applicant-proposing" algorithm offers a transparent and efficient way to match candidates with programs based on preference and availability. Additionally, financial considerations differ, with U.S. medical graduates often burdened with substantial student debt, while Indian medical education, particularly in government institutions, remains highly subsidized. Despite its progress, India's residency admission system still faces significant logistical and administrative challenges, necessitating further reforms such as integrated state and national counseling, real-time seat matrices, and a unified application platform. By examining the structured approach of the U.S. residency match system, India can implement policy changes that improve fairness, efficiency, and transparency in postgraduate medical admissions.

## Introduction and background

The process of getting into residency after medical school is a strenuous one, irrespective of the country in which you graduate. This article attempts to offer a comparative analysis of the entrance examinations and the counseling process for admissions in India versus the United States of America, i.e., the United States Medical Licensing Examination (USMLE) versus the National Eligibility Cum Entrance Test Post Graduate (NEET PG).

India's efforts to bring uniformity to postgraduate (PG) medical counseling have been ongoing for many years, but they have faced numerous legal and political challenges before achieving any significant progress [1]. The first step towards this unification was the administration of the NEET PG exam in 2013, which aimed to streamline the process of medical admissions across the country [1]. Before this, India had a complex and fragmented system of counseling for PG admission, with separate processes for government colleges, private institutions, deemed universities, and central universities, each often governed by its own set of rules. Additionally, institutions like the All India Institute of Medical Sciences (AIIMS), Jawaharlal Institute of Postgraduate Medical Education and Research (JIPMER), and Postgraduate Institute of Medical Education and Research (PGIMER), Chandigarh, conducted their separate counseling, making the entire process confusing and burdensome for students [2].

In contrast, the U.S. follows a structured and uniform residency match process. In the fourth year of medical school, students participate in the National Resident Matching Program (NRMP, started in 1952), a process that begins with the submission of applications in the fall [3]. However, before the main match in March, there are separate matches for certain specialties, such as military residencies, urology, plastic surgery, and ophthalmology, which occur in December or February [4-6]. This system provides a clear, centralized path for medical graduates to transition into their specialty training.

Additionally, in India, the education fees for students studying in government colleges and state quota seats of private medical colleges are significantly subsidized by their respective states. The fees of central government-run medical institutions like AIIMS, PGIMER, and JIPMER are also negligible as the central government funds them entirely. The fees of the remaining students studying in deemed universities and private medical colleges are majorly paid by the parents of the students, greatly minimizing the burden of student loans. This significant difference in funding and infrastructure reflects the complexities of India's medical education system, which still faces challenges in achieving the level of uniformity and transparency seen in more developed systems like the U.S. A stark contrast is seen in the U.S., where the average medical school graduate's debt was about $201,490 in 2019 [7].

## Review

The evolution of PG medical entrance exams in India

Before the introduction of the NEET PG exam in 2013 and the Institute of National Importance Combined Entrance Test (INICET) in 2020, admissions to PG entrance exams used to be conducted through multiple exams [1].

All India Post Graduate Medical Entrance Examination (AIPGMEE)

The AIIMS held a national-level exam for admission to 50% of PG seats in all India quota (AIQ) seats in government medical colleges across India [1].

State-Level Entrance Exams

Different states used to conduct entrance tests for admissions to state government PG seats and private medical college PG seats in that state [1].

Deemed University Entrance Exams

Every deemed university used to conduct its university entrance exam and then do its counseling for the PG seats under its affiliation. It used to be a very opaque process [1].

Institution-Specific Exams

Institutions of national eminence, such as AIIMS, JIPMER, and PGIMER, administered the entrance exams for PG admissions and counseling. Since then, all of the aforementioned tests have been eliminated. In 2013, the AIPGMEE, State Level Entrance Exam, and Deemed University Entrance Exams were combined into the NEET PGexam. In 2020, the AIIMS, JIPMER, and PGIMER Institution-Specific Exams were combined into INICET [2].

The present scenario for PG medical entrance examinations

NEET PG was conducted in 170 cities, and 228,540 candidates appeared for some 61,789 seats available in Doctor of Medicine (MD), Master of Surgery (MS), Postgraduate Diploma, and Diplomate of National Board (DNB) seats [8,9]. Previously, MD/MS counseling was separate from DNB counseling, but they have been combined for the last two to three years [10]. Two different types of authorities conduct the counseling [10]. One is the Medical Counselling Committee (MCC), which performs all India counselling, and the second is state counselling authorities, which are different in every state [10]. Before the counseling process starts, each college shares the availability of recognized seats with state and center authorities [10]. In government PG, seats are divided into 50% quotas: AIQ seats and state quota seats [10]. Candidates domiciled residents of a particular state are eligible for that state's quota seats (50%) [10].

Categories of seats allocated by the MCC

The MCC is responsible for the allocation of the following categories of seats:

(a) 50% of the AIQ seats from all states, with the inclusion of the Union Territory of Jammu and Kashmir contingent upon their contribution of seats [11].

(b) 100% of seats in deemed universities.

(c) 50% of PG AIQ seats in colleges under the Employee State Insurance Corporation (ESIC) are reserved for wards of insured individuals [11].

(d) All PG seats in Armed Forces Medical Services Institutions and specific central institutes, including Vardhman Mahavir Medical College (VMMC) & Safdarjung Hospital, Atal Bihari Vajpayee Institute of Medical Sciences (ABVIMS) & Dr. Ram Manohar Lohia (RML) Hospital, and ESIC Institute PGIMSR, Basaidarapur. These seats are divided into 50% AIQ seats and 50% under Indraprastha (I.P.) University [11].

(e) 100% of seats in Central Universities, such as Aligarh Muslim University, Banaras Hindu University, the University of Delhi, and other central institutes, are subject to eligibility criteria detailed in the MCC counseling scheme [11].

Next, students must rank their desired choices for PG seats in order of preference from most desired to least desired alternatives from top to bottom [10]. Each student's choices are matched to the available seat based on their NEET PG entrance exam rank [10].

The counseling process for seats on the MCC is divided into several stages, including the first, second, third, mop-up, and final stray vacancy rounds [10]. State-level counseling follows a similar process: first, second, third, mop-up, and final stray vacancy rounds. The state counseling rounds occur after the corresponding MCC rounds are completed [10].

India also adopted Diversity Equity criteria for inclusion and upliftment of various strata of society. As a part of this, the eligibility criteria vary for different categories of students. The eligibility to attend either counseling is based on the minimum qualifying/eligibility criteria for admission as mentioned in the Information Bulletin for NEET PG as shown in Table 1 [12].

**Table 1:** Minimum Qualifying Percentile Criteria for NEET PG Counseling Based on Candidate Category This table presents the minimum qualifying percentile criteria for NEET PG counseling across various candidate categories. The eligibility thresholds differ for General, EWS, PwBD, SC, ST, and OBC applicants. Historically, cutoff percentiles have often been revised following initial counseling rounds. In 2023, the cutoff was controversially lowered to 0 percentile, while in 2024, it was adjusted to 15 percentile for General candidates and 10 percentile for reserved categories, reflecting ongoing changes in medical admission policies. In 2025, the cutoff was further reduced to 5% for all categories. NEET PG: National Eligibility Cum Entrance Test Post Graduate

Category	Minimum Qualifying/Eligibility Criteria
General/EWS (Economically Weaker Section)	50th Percentile
General-PwBD (Persons with Benchmark Disabilities)	45th Percentile
SC/ST/OBC (Scheduled Caste/Scheduled Tribe/Other Backward Caste, including PwBD of SC/ST/OBC)	40th Percentile

The eligibility criteria to clear USMLE steps are shown in Table 2.

**Table 2:** Minimum Qualifying Criteria for Medical Residency Admissions in the USA This table displays the minimum passing scores for the USMLE examinations. Note that USMLE Step 1 and Step 2 CK are required for admission into residency programs. Although USMLE Step 3 is not needed to start residency, it is mandatory to graduate from residency and to obtain a medical license in the United States. USMLE: United States Medical Licensing Examination

Exam	Minimum Passing Score
The passing score for the USMLE Step 1	196
The minimum passing score for the USMLE Step 2 Clinical Knowledge (CK) exam	214
The minimum passing score for the USMLE Step 3 exam	200

In the past, the National Board of Examination (NBE) has dropped the cutoff from 50 percentile to 40, 30, or 20 after two rounds of counseling, but in 2023, it was reduced to 0 percentile [10]. Following public criticism in 2024, the eligibility percentiles were reduced to 15 for general categories and 10 in reserved categories, rather than 0 in 2023 [13]. The tentative counseling schedule for the year 2024 is shown in Table 3 [14].

**Table 3:** Tentative Counseling Schedule for NEET PG 2024 Admissions This table presents the tentative counseling schedule for NEET PG 2024 admissions, outlining the key rounds of seat allocation, data sharing between central and state authorities, and final joining deadlines. The counseling process was initially planned to conclude by January 2025; however, due to administrative delays, legal challenges, and seat reallocation issues, the process remained unfinished even by March 2025, causing significant uncertainty for medical graduates awaiting residency placements. NEET PG: National Eligibility Cum Entrance Test Post Graduate; MCC: Medical Counselling Committee; DME: Directorate of Medical Education

S. No.	Admissions Schedule	All India Quota/Deemed and Central Universities	MCC's Sharing of Joined Candidate Data	State Counselling	Data Sharing for Joined Candidates by State Counseling Authorities and DMEs
1.	1st round of counselling	September 20, 2024, to November 20, 2024	November 28, 2024, to November 29, 2024	November 18, 2024, to November 27, 2024	December 5, 2024, to December 6, 2024
2.	The last day to join	November 27, 2024	-	December 4, 2024	-
3.	2nd round of counselling	December 4, 2024, to December 12, 2024	December 21, 2024, to December 22, 2024	December 12, 2024, to December 23, 2024	December 29, 2024, to December 31, 2024
4.	The last day to join	December 20, 2024	-	December 28, 2024	-
5	Round-3	December 26, 2024, to January 1, 2025	December 26, 2024, to January 1, 2025	January 7, 2025, to January 13, 2025	January 19, 2025, to January 20, 2025
6	The last day to join	January 13, 2025	-	January 18, 2025	-
7	Stray vacancy	January 18, 2025, to January 24, 2025	January 31, 2025	January 25, 2025, to January 30, 2025	-
8	The last day to join	January 30, 2025	-	February 5, 2025	-
9	Academic session for PG courses begins	December 20, 2024	-	December 20, 2024	-

Students benefited greatly from these changes in the PG entrance process because earlier, they had to register for multiple examinations, travel to various exam locations, and deal with numerous scheduling difficulties that led to financial and psychological strain.

Match process in the U.S.

There are four different counselors for residency in the USA: NRMP Match (started in 1952), San Francisco Match (began in 1979), Urology Match (started in 1985), and Military Match (began in 1952).

NRMP Match

In 1952, at the request of medical students, the NRMP was created to offer a systematic and equitable way to match the preferences of residency program directors with those of applicants for U.S. residency seats. The NRMP oversees the Specialities Matching Service® (SMS®) for advanced fellowship training and the Main Residency Match® for core residency training in almost all recognized specialties [3]. The 2025 schedule is shown in Table 4 [15].

**Table 4:** 2025 NRMP Match Schedule: Timeline for U.S. Medical Residency Placement This table provides the 2025 NRMP Match schedule, detailing key dates for the U.S. residency application and selection process. The timeline includes important milestones such as registration opening, rank order list submission, the Supplemental Offer and Acceptance Program (SOAP), and the Match Day results announcement. The NRMP Match follows an algorithm-driven system to allocate residency positions fairly based on applicant preferences and program selections. Unlike India's NEET PG counseling, which faced delays extending into March 2025, the U.S. residency match adheres to a structured and timely process, ensuring that medical graduates transition smoothly into residency programs starting in July. NRMP: National Resident Matching Program; NEET PG: National Eligibility Cum Entrance Test Post Graduate

Date	Event
September 16	Match Registration Opens: The candidate can build their R3 account after registration opens. The candidate must register for both the NRMP and the application service or procedure that the program requires. The deadline for Standard Registration is January 31, 2025.
February 03	Ranking Opens: To create and modify their Rank Order List, log in to R3. Each program's finalized quota of posts is available for examination in the Program Directory. Medical schools start checking the qualifications of students and graduates.
March 05	Deadline for Rank Order List Certification: Verification deadline for medical school students and graduates’ qualifications. Deadline for late registration for the match, SOAP, and match withdrawal deadline for applicants.
March 17	The status of the applicant match is available. If matched, the SOAP notifies applicants. The List of Unfilled Programs is accessible to SOAP-eligible unmatched and partially matched candidates.
March 18	Programs start going over SOAP apps. After receiving an application, programs may contact candidates and start the interview process.
March 20	Round 1 of SOAP: Applicants receive offers for Round 1. The deadline for applicants to accept or reject offers from Round 1. Round 2 of SOAP: Offers for Round 2 are sent to applicants. The deadline for applicants to accept or reject offers from Round 2. Round 3 of SOAP: Round 3 offers are sent to applicants. The deadline for applicants to accept or reject Round 3 offers. Round 4 of SOAP: Round 4 offers are sent to applicants. The deadline for applicants to accept or reject Round 4 offers. SOAP Ends: Candidates can start contacting all of the open programs. As vacancies are filled, programs can update the List of Unfilled Programs.
March 21	The Match Day Applicant: Match results are accessible via email and the R3 system. Match day ceremonies for medical school.

San Francisco Match

It only offers counseling for ophthalmology and plastic surgery residency programs [4]. Its schedule is shown in Table 5 [16].

**Table 5:** 2025 San Francisco Match Schedule This table outlines the 2025 San Francisco Match schedule, which is used for residency placements in ophthalmology and plastic surgery in the United States. The timeline includes key milestones such as applicant registration, interview periods, rank list submission deadlines, and match result announcements. Unlike the NRMP Match, the San Francisco Match operates independently and follows an earlier timeline, with results typically released in February. NRMP: National Resident Matching Program; PT: Position Tracker; CAS: Centralized Application Service

Date	Event
July 1, 2024: Applicant Registration	Registration for applicants opens. Registration remains open until the rank list deadline; however, applicants are strongly advised to register early to ensure sufficient time for the application process and interviews.
CAS Target Date: September 3, 2024	Applications are made available to programs. Applicants can upload supplemental documents and additional applications as they become available. This is NOT a deadline, and programs may continue accepting applications beyond this date. It is the applicant's responsibility to check with individual training programs for their specific deadlines.
July 1, 2024, through January 25, 2025, Program-driven Open House setup	Open House setup period for programs.
Medical School Performance Evaluation (MSPE) letters on September 25, 2024	MSPE letters are made available to programs.
Interview Invitation Period: October 8, 2024-December 2, 2024	The first and final dates on which interviews may be extended.
Interview Period: October 22-December 20, 2024	Interview period: All interviews will be conducted virtually.
October 23, 2024, to January 25, 2025, Saturday Open House: time for applicants to sign up	The registration period for applicants to sign up for Open House tours.
January 3, 2025, Deadline for the Program Rank List	Deadline for program rank list submission: All programs must submit their rank lists.
January 4, 2025, to January 25, 2025, Tours of Open Houses	Voluntary program visits.
January 28, 2025, Deadline for the Applicant Rank List	Deadline for applicant rank list submission: All applicants must submit their rank lists.
Results of the match on February 4, 2025	Match results are accessible to programs, applicants, and U.S. medical schools via the San Francisco Match system. A login is required to view the results.
February 4, 2025, Post-match PT openings	Any post-match vacancies will be posted on the Immediate Vacancies page. Individual programs are responsible for managing their vacancy listings.
June 2025-July 2026: Start of Training	PGY-1 residency training commences.

Urology Match

The American Urology Association (AUA) and the Society of Academic Urologists have managed the Urology Residency Match Program (also known as the Urology Match) for residency places for over 39 years. Every year, over 500 highly competitive candidates apply for the nearly 385 seats that are essentially filled through the Urology Residency Match [5]. It happens in the format given in Table 6 [17].

**Table 6:** 2025 Urology Residency Match Schedule: Key Dates and Application Process This table details the 2025 Urology Residency Match schedule, which is overseen by the American Urological Association (AUA) and the Society of Academic Urologists. The timeline includes critical steps such as registration, application signaling, interview offers, preference list submission, and final match results announcement. Unlike the NRMP Match, the Urology Match operates independently with an earlier schedule, ensuring that successful candidates receive their placements by February 2025. NRMP: National Resident Matching Program

Date	Event
June 2, 2024	Urology registration opens for programs and applicants
September 2, 2024	Applicants signaling opens
September 16, 2024	Applicants' deadline to submit signals
September 25, 2024	Applicant signals sent to programs
October 25, 2024	All programs offer interview
October 28, 2024	All applicants accept or reject interview offers
October 29, 2024	"Flush Day" students may release previously accepted interview slots in favor of any invitations offered to them off a waitlist
November 21, 2024	Urology preference list phase begins for programs and applicants
December 27, 2024	Registration deadline
January 6, 2025	Urology preference list deadline (program and applicants) and interview deadline
February 3, 2025	Urology Match results announced; emails sent and results posted

Military Match

The Military Match is quite similar to the civilian match, with a few key differences depending on the branch of the military you are in. The Military Match occurs earlier, in December, rather than in March. As a result, the schedule has been pushed forward slightly. The application process may also differ. Beginning in 2019, PGY-1 students will submit documentation using a paper application rather than the Medical Operational Data System (MODS) used by the Air Force, Army, and Navy. The application, like the civilian match, contains a curriculum vitae, personal statement, USMLE scores, transcript, Medical Student Performance Evaluation (MSPE), and letters of recommendation. The branch will provide candidates with instructions on how to upload them. The Joint Service Graduate Medical Education (JSGME) Board rates applicants in mid-November using criteria such as grades, USMLE scores, research, "audition" rotations, interviews, and past military service. Candidates can be assigned to either their primary military residency, a secondary specialty, a civilian residency (only for the Air Force and Navy), or a military transitional year. The Army, Navy, and Air Force have their own match process [6].

The NRMP Match algorithm

How Does This Work?

The matching algorithm is "applicant-proposing," which means it tries to place an applicant (Applicant A) in the program that appears to be the most preferred on Applicant A's ranking list. Suppose Applicant A cannot be matched to this first-choice program (because the program does not prefer Applicant A), in that case, an attempt is made to place Applicant A in the second choice program, and so on, until Applicant A obtains a tentative match, or all of Applicant A's options have been exhausted [18].

What Does "Tentative Match" Mean?

Applicant A will be tentatively matched to a program if it ranks them on its rank order list and either has an open position or prefers Applicant A over another applicant (Applicant B) who has already been tentatively matched. In this situation, Applicant B is "bumped" from the tentative match with the program to make way for Applicant A [18].

What Happens When an Applicant's Tentative Match Is "Bumped"?

The matching algorithm will return to Applicant B's rank order list and make a preliminary match at the next most preferred place on Applicant B's list. The same steps are taken to identify another tentative match for Applicant B as for Applicant A [18].

When Does a Tentative Match Become Final?

When all applicants' rank order lists have been considered, the matching algorithm is finished, and all tentative matches are final and binding for training [18].

Discussion

India is moving in the right direction. Earlier, it used to have multiple entrances. Each state used to have one, each deemed university used to have one, and each institute of national eminence used to have one. There was also one national exam for AIQ seats.

After so many obstacles, India moved on to a single NEET PG exam for all the PG seats except those of institutes of national eminence. The current structure and implementation of medical counseling for PG admissions in India, particularly under the AIQ and state counseling processes, are relatively inefficient and inconsistent, resulting in a lack of transparency, fairness, and synchronization. These challenges manifest as follows:

Seat Blocking and Resource Misallocation

Candidates compete in AIQ and state counseling rounds, frequently blocking seats without a genuine desire to get into that specific college. This results in mid-ranking candidates losing opportunities to acquire their preferred seats, while lower-ranking candidates profit disproportionately from last-minute seat availability during mop-up or stray rounds.

Asynchronous Counseling Rounds

The absence of coordination between AIQ and state counseling schedules causes candidates to judge based on inadequate or ambiguous information, resulting in suboptimal outcomes. Candidates frequently block AIQ seats out of caution, reducing the availability of seats for others in the following rounds. Then, they often resign AIQ seats for state quota seats. But, when the resigned seat becomes available for counseling, students who are immediately next in line and would have been eligible for the seat become ineligible for those seats.

Judicial Interventions and Delays

Frequent lawsuits involving unresolved counseling issues disrupt the process, producing delays and confusion for candidates.

Mid-counselling Changes in Seat Availability

This system becomes unfair when seats are added or deleted in subsequent rounds, which affects candidates who have already decided based on previous allocations. Inequitable seat distribution, extreme psychological strain, and candidate discontent are all caused by these structural problems. Many students struggle to get seats that suit their preferences despite their hard work and high rankings because of poor administration, a lack of coordination, and an excessive dependence on happenstance. Resolving these issues is essential to guaranteeing an open, equitable, and effective counseling procedure that respects the constitutional reservation and merit standards.

Possible solutions to overcome the above challenges

Accountability

The procedures should be held accountable, and the counseling process as a whole should be done following a scheduled and well-planned timeline without any delays or postponements.

Release an Ideal Seat Matrix Before Counseling

This guarantees that there won't be any seat confusion between rounds. Some seats are added unexpectedly in round 2, and individuals who retained their seats in round 1 do not receive the chance to apply for those seats, perplexing others who had applied for an upgrade.

Integrated All India and State Counseling

MCC and state counseling must occur simultaneously to avoid time gaps. This will prevent seat blocking and offer everyone an equal chance to obtain the available seat at their level.

Unified Platform for All India and State Counselling

Multiple states and all-India counseling are highly difficult for students to follow. Hence, it should be done on a single platform like MCC. It would make it easier for students to apply to several states.

Conducting Offline/In-Person Counselling (Combined for All India and State)

We can offer rank-based offline counseling based on the seats the student qualifies for based on the state quota and any reservations. As a result, seats will be taken on the spot and won't be blocked. No time is wasted on state counseling, resignations from seats due to state counseling, or document verification. As a result, the report time currently granted after each round will be reduced by seven days.

In the US, medical students start applications for residency in July, and the first match comes in December, which is the Military Match, and the next match, whose results are the Urology and San Francisco Match. San Francisco Match is for ophthalmology and plastic surgery only [6,16,17]. The main match is the NRMP Match, whose results come in mid-March. There is a week for the Supplemental Offer and Acceptance Program (SOAP) Match for the students who did not match in the main match [15]. Then, the NRMP Match results are declared, the students are matched, and the residency training program starts in July [15]. This leads to minimal wastage of time for the medical students between their medical school and residency.

In India, the NEET PG exams are at the end of medical school, and the whole counseling procedure starts after the exam results are declared. Table 7 gives tentative timelines for the entire admission process to residency in India compared to the U.S. as of 2026 [14,15].

**Table 7:** Comparison of Residency Admission Timelines: NEET PG (India) vs. NRMP Match (USA) NEET PG: National Eligibility Cum Entrance Test Post Graduate; NRMP: National Resident Matching Program; SOAP: Supplemental Offer and Acceptance Program

India	USA
Conduction of NEET PG Examination after completion of five and a half years of medical school on August 11, 2024.	Completion of Step 1 examination after the second year (of 4-year program) of medical school. Completion of Step 2 examination after the third year of medical school.
Commencement of 1st round of counseling for admission into residency on September 20, 2024.	Registration for the match cycle is on September 16, 2024.
2nd round of counselling from December 4, 2024.	Rank order on February 3, 2025.
3rd round from December 26, 2024.	Rank order list deadline on March 3, 2025
Stray vacancy from January 18, 2025.	Application Match status available on March 17, 2025
Last date of joining: January 30, 2025.	The programs begin reviewing SOAP applications on March 18, 2025
-	SOAP rounds 1, 2, 3, and 4 on March 20, 2025
-	Match day on March 21, 2025

The pros and cons of the NEET PG examination and the USMLE

NEET PG

A rigorous approach that guarantees consistency but places great strain on one test, conducted once a year. No other parameters are taken into consideration. It is most appropriate for students who perform well under pressure.

USMLE

A more adaptable system that permits flexibility (the written tests are conducted throughout the year), but at the price of possible postponement and increased costs. It uses a more holistic selection process based on multiple parameters, including three written examinations, clinical experience, interviews, extracurricular activities, research publications, etc. Perfect for applicants who appreciate flexibility and are well-rounded.

## Conclusions

The introduction of NEET-PG has unified India's PG medical admissions, simplifying a previously fragmented system of multiple state-level and institution-specific exams. This consolidation has reduced logistical, financial, and psychological burdens on medical aspirants, enabling a more standardized and equitable admission process. However, challenges persist, particularly in the counseling process. Asynchronous AIQ and state counseling, seat blocking, mid-counseling seat changes, and judicial interventions undermine fairness and efficiency. Proposed solutions, including an integrated counseling platform, simultaneous AIQ and state rounds, and pre-published seat matrices, could address these inefficiencies and improve transparency.

In contrast, PG medical residency counseling in the U.S., such as the NRMP Match, San Francisco Match, Urology Match, and Military Match, offers a structured and transparent algorithm-based system. The "applicant-proposing" algorithm ensures fairness by aligning applicant preferences with program selections, minimizing disruptions like seat blocking. Lessons from the USA's structured counseling system, such as synchronized schedules, centralized platforms, and binding rank-order algorithms, can inspire reforms in India's counseling framework. By addressing the existing inefficiencies and adopting proven practices from systems like the NRMP, India's PG medical admissions process can be more streamlined, equitable, and efficient, ensuring the best outcomes for candidates and institutions.
